# Can dosimetric parameters predict acute hematologic toxicity in rectal cancer patients treated with intensity-modulated pelvic radiotherapy?

**DOI:** 10.1186/s13014-015-0454-0

**Published:** 2015-08-04

**Authors:** Juefeng Wan, Kaitai Liu, Kaixuan Li, Guichao Li, Zhen Zhang

**Affiliations:** Department of Radiation Oncology, Fudan University Shanghai Cancer Center; Department of Oncology, Shanghai Medical College, Fudan University, Shanghai, Shanghai, China; Department of Radiation Oncology, Lihuili Hospital, Ningbo Medical Center, Ningbo, 315041 China; Department of Radiation Oncology, Fudan University Shanghai Cancer Center, 270 Dong An Road, Shanghai, 200032 China

**Keywords:** Dosimetric parameters, Hematologic toxicity, Rectal cancer, Pelvic radiotherapy

## Abstract

**Background:**

To identify dosimetric parameters associated with acute hematologic toxicity (HT) in rectal cancer patients undergoing concurrent chemotherapy and intensity-modulated pelvic radiotherapy.

**Methods:**

Ninety-three rectal cancer patients receiving concurrent capecitabine and pelvic intensity-modulated radiation therapy (IMRT) were analyzed. Pelvic bone marrow (PBM) was contoured for each patient and divided into three subsites: lumbosacral spine (LSS), ilium, and lower pelvis (LP). The volume of each site receiving 5–40 Gy (V 5, V10, V15, V20, V30, and V40, respectively) as well as patient baseline clinical characteristics was calculated. The endpoint for hematologic toxicity was grade ≥ 2 (HT2+) leukopenia, neutropenia, anemia or thrombocytopenia. Logistic regression was used to analyze correlation between dosimetric parameters and grade ≥ 2 hematologic toxicity.

**Results:**

Twenty-four in ninety-three patients experienced grade ≥ 2 hematologic toxicity. Only the dosimetric parameter V40 of lumbosacral spine was correlated with grade ≥ 2 hematologic toxicity. Increased pelvic lumbosacral spine V40 (LSS-V40) was associated with an increased grade ≥ 2 hematologic toxicity (*p* = 0.041). Patients with LSS-V40 ≥ 60 % had higher rates of grade ≥ 2 hematologic toxicity than did patients with lumbosacral spine V40 < 60 % (38.3 %, 18/47 vs.13 %, 6/46, *p* =0.005). On univariate and multivariate logistic regression analysis, lumbosacral spine V40 and gender was also the variable associated with grade ≥ 2 hematologic toxicity. Female patients were observed more likely to have grade ≥ 2 hematologic toxicity than male ones (46.9 %, 15/32 vs 14.8 %, 9/61, *p* =0.001).

**Conclusions:**

Lumbosacral spine -V40 was associated with clinically significant grade ≥ 2 hematologic toxicity. Keeping the lumbosacral spine -V40 < 60 % was associated with a 13 % risk of grade ≥ 2 hematologic toxicity in rectal cancer patients undergoing concurrent chemoradiotherapy.

## Introduction

Preoperative chemoradiotherapy (CRT) followed by total mesorectal excision is the standard of care for patients with locally advanced rectal cancer (LARC) [3, 10, 19, 20]. The delivery of 5-Fluorouracil (5-FU) based chemotherapy with radiotherapy reduces 5-year incidence of local recurrence compared with radiotherapy (RT) alone [11]. Park *et al*. demonstrated tumor response to neoadjuvant CRT was associated with 5-year recurrence free survival (RFS) [18]. However, myelosuppression is a major common side effect of CRT that could lead to treatment interruptions [9, 25]. Thus, the reduction of hematologic toxicity (HT) is an important goal.

Pelvic radiotherapy may contribute to the development of HT. More than one-half of the body’s bone marrow is located in the os coxae, sacrum, proximal femora, and lower lumbar spine. Therefore, reducing pelvic bone marrow (PBM) irradiation may reduce HT, enabling improved delivery of chemotherapy, and, consequently, treatment efficacy.

Several studies demonstrated a correlation between PBM dosimetric parameters with HT in patients with anal cancer and cervical cancer. Thus, recommended dose constraints to the LSS are V10 ≤ 80 %, and keeping the mean PBM dose < 22.5 Gy and <25 Gy is associated with a 5 % and 10 % risk of HT, respectively, in patients with anal cancer [5, 7]. Data from cervical cancer suggest that patients with PBM-V10 ≥ 90 % had higher rates of Grade 2 HT than did patients with PBM-V10 < 90 % [16].

The clinical significance and optimal technique of PBM sparing in rectal cancer patients, however, are still unknown. Here, we set out to identify PBM dosimetric parameters that correlate with HT in patients treated with CRT for rectal cancer.

## Materials and methods

We conducted a retrospective review of 93 patients with LARC (cT3-T4 and/or cN+) who were treated with neoadjuvant CRT at our institution between September 2013 and August 2014. The study was approved by the Fudan University Shanghai Cancer Center Institutional Review Board.

### Combined chemoradiotherapy

#### Radiotherapy

**Fig. 1:**
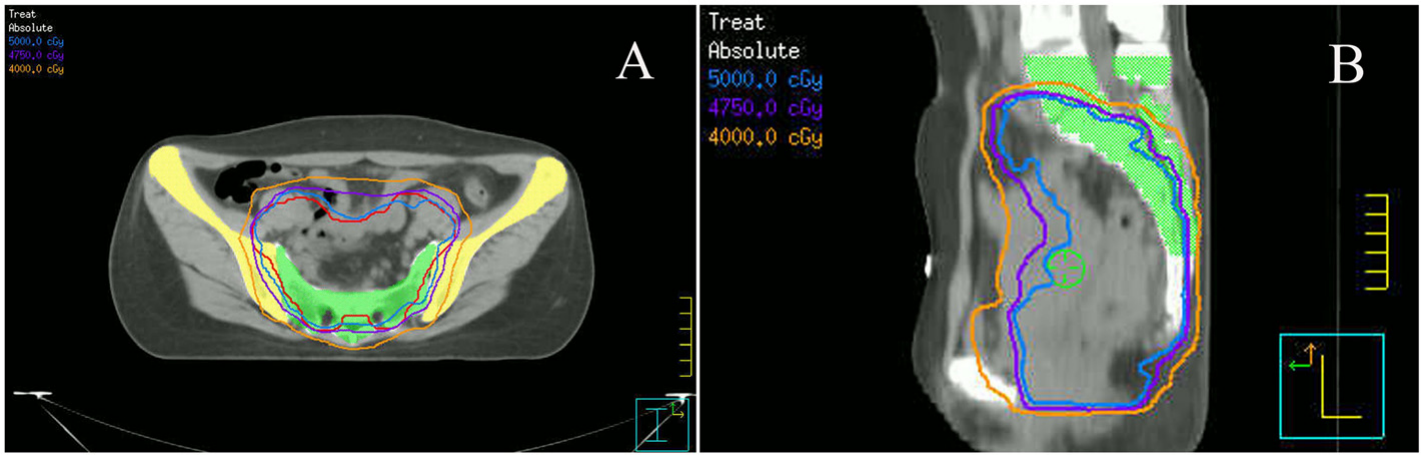
IMRT isodose distribution of representative axial (**a**) and sagittal (**b**) slice. PTV is shown in red. Blue, purple, and aurantium isodose lines represent 50, 47.5, and 40 Gy, respectively

Patients were immobilized in the prone or supine position and underwent a non-contrast planning CT scan with a 5-mm slices from the L3-L4 junction to 2 cm below the perineum. The image datasets were transferred to the PINNACLE planning system (Philips Radiation Oncology Systems, Milpitas, CA). The gross tumor volume (GTV) was defined as all known gross disease determined from CT and MRI. The clinical target volume (CTV) was defined as the GTV plus areas considered at significant risk of harboring microscopic disease, including the mesorectum (perirectal fascia), presacral region, and internal iliac lymph node region. Based on our institution set-up data, the planning target volume (PTV) was generated by adding a 6-mm margin around the CTV in lateral and anterior-posterior directions, and an 8-mm margin in the superiorinferior direction [26] (Fig. 1). The critical normal organs at risk (OARs) outlined were the bladder, femoral heads, and small bowel.

The PTV was prescribed with a total of 50 Gy in 2Gy fractions. The intensity-modulated radiation therapy (IMRT) plans were generated using the inverse planning module of PINNACLE for a 6-MV linear accelerator, with seven coplanar fields. The D2 %, D50 %, and D98 % to PTV were set at 52.5 Gy, 50 Gy and 47.5 Gy, respectively. The dose of the OARs was set as low as possible and had to at least meet the following constraints: bladder, V45 ≤ 15 % and V40 ≤ 40 %; femoral heads, V45 ≤ 25 % and V40 ≤ 40 %; small bowel, V45 ≤ 65 cc, V40 ≤ 100 cc, and V35 ≤ 180 cc.

### Concurrent chemotherapy

Capecitabine combined was administered concurrently with pelvic radiation. Capecitabine was given at a dose of 825 mg/m2 twice daily from Monday to Friday throughout the whole course of IMRT. Guidelines for capecitabine usage were as follows: for grade ≥ 2 toxicity (as defined by NCI CTCAE version 4.0), capecitabine was held, and appropriate symptomatic treatment was administered. Once toxicity resolved to grade 0 or 1, treatment was resumed.

### Pelvic bone marrow delineation

**Fig. 2:**
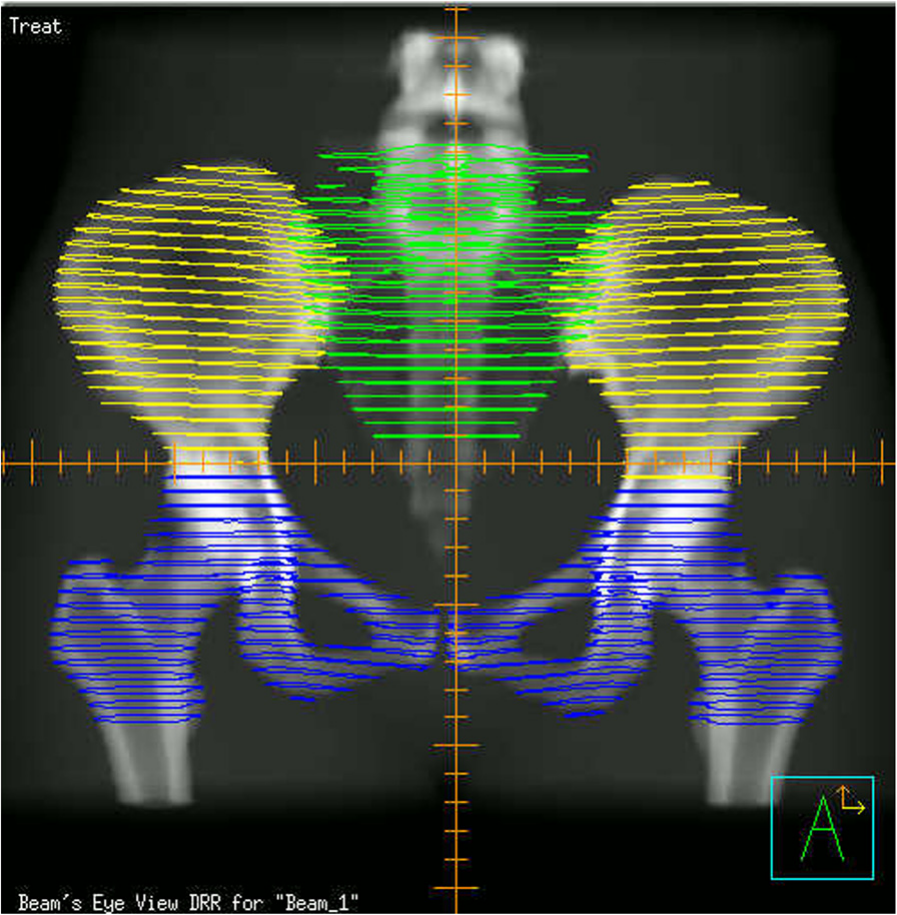
Coronal section illustrating delineation of iliac (*yellow*), lumbosacral (*green*), and lower pelvic (*blue*) bone marrow

For each patient, the external contour of all bones within the pelvis was used as a surrogate for PBM, and the PBM was further divided into three subsites, as described by Mell *et al.*: (1) ilium—including the iliac crests extending to the superior border of the femoral heads; (2) lower pelvis (LP)—consisting of the pubes, ischia, acetabula, and proximal femora, extending from the superior border of the femoral heads to the inferior border of the ischial tuberosities; and (3) lumbosacral spine (LSS)—extending from the most superior vertebral body contained in the planning treatment volume (usually L5) inferiorly to include the entire sacrum [16] (Fig. 2). Dose-volume Histograms (DVHs) were then generated, and the following parameters were recorded for the PBM and each subsite: volume, mean dose, and volume of each region receiving at least 5, 10, 15, 20, 30, and 40 Gy (V5, V10, V15, V20, V30, and V40, respectively).

### Hematologic toxicity

The HT was graded according to the Common Terminology Criteria for Adverse Events, version 4.0. The highest-grade toxicity for white blood count, absolute neutrophil count, hemoglobin, and platelets were recorded, with HT of grade ≥2 noted as an event (HT2+).

### Statistical analysis

Age and dosimetric parameters were coded as continuous variables. Categorical variables included gender. Univariate logistic regression was used to test the correlation between clinical and dosimetric parameters with HT2+. Multivariate logistic regression models were then used to examine the effect of significant dosimetric parameters on HT2+. The incidence of HT2+ between male and female was compared by Pearson’s chi-square test.

## Results

### Patient characteristics

Baseline characteristics of the cohort are shown in Table 1. The median age at diagnosis was 57 years, with a male predominance. The majority of patients had clinical stage III tumors (79.6 %).

**Table 1:** Patient characteristics (*n* = 93)

Characteristic	No. of patients (%)
Gender	
Male	61(65.6)
Female	32(34.4)
Age in yrs	
Mean	54.3
Median	57
Range	30–73
Clinical stage	
II	19(20.4)
III	74(79.6)

### Bone marrow dosimetric parameters

Table 2 summarizes the PBM dosimetric parameters. The median PBM mean dose was 29 Gy. The LSS was the smallest subsite of the PBM (22 %), and the LP was the largest subsite (46 %).

**Table 2:** Descriptive statistics of pelvic bone marrow dosimetric parameters

Parameter	Median value (range)
Pelvic bone marrow	
Volume (mL)	1311(882-1663)
Mean dose (cGy)	2944(2402-3410)
V5 (%)	93(82-100)
V10 (%)	86(73-99)
V15 (%)	81(66-97)
V20 (%)	72(56-84)
V30 (%)	48(34-61)
V40 (%)	31(18-42)
Lumbosacral spine	
Volume (mL)	285(213-387)
Mean dose (cGy)	3616(2505-4550)
V5 (%)	89(64-100)
V10 (%)	81(55-100)
V15 (%)	78(53-100)
V20 (%)	75(44-100)
V30 (%)	70(47-96)
V40 (%)	60(37-77)
Ilium	
Volume (mL)	421(283-549)
Mean dose (cGy)	2797(2233-3546)
V5 (%)	90(77-100)
V10 (%)	82(66-99)
V15 (%)	77(61-99)
V20 (%)	68(54-93)
V30 (%)	46(30-73)
V40 (%)	26(14-42)
Low pelvis	
Volume (mL)	593(385-808)
Mean dose (cGy)	2748(1928-3893)
V5 (%)	97(78-100)
V10 (%)	91(70-100)
V15 (%)	85(56-99)
V20 (%)	69(43-93)
V30 (%)	38(16-70)
V40 (%)	18(6-41)

### Hematologic toxicity

Overall, 51 patients (51/93, 54.8 %) experienced leukopenia during treatment. The percentage of patients developing acute neutropenia, anemia, and thrombocytopenia was 25.8 %, 6.5 %, and 6.5 %, respectively. The percentage of patients with grade 2 or worse leukopenia, neutropenia, anemia, and thrombocytopenia was 25.8 %, 16.1 %, 2.2 %, and 1.1 %, respectively (Table 3). 24 patients (24/93, 25.8 %) experienced HT2+ during chemoradiotherapy. 21 patients (21/24, 87.5 %) experienced only grade 2 or worse leukopenia and/or neutropenia. 2 patients experienced (2/24, 8.3 %) both grade 2 or worse leukopenia/ neutropenia and anemia and 1 patients (1/24, 4.2 %) experienced both grade 2 or worse leukopenia and thrombocytopenia. HT2+ was frequently observed in females (male/female: 46.9/14.8 %) (*p*-value =0.001) (Table 4).

**Table 3:** Acute hematologic toxicity during chemoradiotherapy

Toxicity	Grade 0	Grade 1	Grade 2	Grade 3
Leukopenia	42(45.2)	27(29)	21(22.6)	3(3.2)
Neutropenia	69(74.2)	9(9.7)	12(12.9)	3(3.2)
Anemia	87(93.5)	4(4.3)	1(1.1)	1(1.1)
Thrombocytopenia	87(93.5)	5(5.4)	1(1.1)	0(0)

**Table 4:** Acute HT2+ during chemoradiotherapy

Characteristic	HT2+
Leukopenia and/or neutropenia	21(21/24, 87.5 %)
Leukopenia and/or neutropenia + anemia	2 (2/24, 8.3 %)
Leukopenia and/or neutropenia + thrombocytopenia	1(1/24, 4.2 %)
Gender	
Male	9(9/61, 14.8 %)
Female	15(15/32, 46.9 %)

### Predictors of hematologic toxicity

On univariate analysis, LSS-V40 and gender were associated with HT2+ (Table 5). Patients with LSS-V40 ≥ 60 % had higher rates of HT2+ than did patients with LSS-V40 < 60 % (38.3 %, 18/47 vs.13 %, 6/46, *p* =0.005). The incidence of HT2+ was higher in female patients (46.9 %, 15/32) than in male patients (14.8 %, 9/61) (*p*-value =0.001). On multivariate analysis, LSS-V40 and gender retained statistical significance (Table 6).

**Table 5:** Univariate logistic regression analysis of factors associated with the development of HT2+

Parameter	*P* value	Odds ratio
Age	0.162	1.038
Gender	0.005	6.5
Pelvic bone marrow		
Mean dose	0.735	1.00
V5	0.227	1.1017
V10	0.279	1.064
V15	0.633	1.023
V20	0.875	0.993
V30	0.848	0.99
V40	0.764	1.018
Lumbosacral spine		
Mean dose	0.306	1.001
V5	0.712	1.017
V10	0.475	0.031
V15	0.462	1.031
V20	0.425	1.031
V30	0.406	1.035
V40	0.041	1.09
Ilium		
Mean dose	0.434	1.001
V5	0.115	1.092
V10	0.099	1.074
V15	0.261	1.045
V20	0.642	1.017
V30	0.921	1.004
V40	0.742	1.017
Low pelvis		
Mean dose	0.734	1.00
V5	0.654	1.028
V10	0.805	1.009
V15	0.759	0.991
V20	0.642	1.017
V30	0.921	1.004
V40	0.314	0.948

**Table 6:** Multivariate logistic regression analysis

Parameter	*P* value	Odds ratio
Age	0.57	0.913
Gender	0.017	6.581
Lumbosacral spine		
V40	0.035	1.052
Ilium		
V5	0.602	0.901
V10	0.356	1.166

## Discussion

To our knowledge, this is the second study to predict acute HT in patients with rectal cancer receiving CRT. *Yang TJ et al.* found that coxal BM V45 and sacral BM V45 were associated with lower WBC and ANC nadirs [25]. In this study we demonstrated that LSS-V40 was associated with the development of HT2+ following chemoradiation to the pelvic. Additionally, HT2+ was more likely to occur in women.

There have been several studies investigating the dosimetric parameters of PBM that correlate with the risk of HT on cervical and anal cancer [1, 4, 7, 16, 21]. Mell *et al.* found patients with PBM-V10 ≥ 90 % had higher rates of Grade 2 or worse leukopenia and neutropenia than did patients with BM-V10 < 90 % [16]. Rose *et al.* found V10 ≥ 95 % were more likely to experience Grade ≥ 3 leukopenia than were patients with V20 > 76 % in a similar patient cohort [21]. Albuquerque *et al.* studied 40 women who received CRT to treat cervical cancer and found the risk of HT2+ developing increases by a factor (odds ratio) of 4.5 if the V20 of the whole pelvis exceeds 80 % [1]. For patients with anal cancer, Cheng *et al.* demonstrated mean dose and low-dose radiation parameters (V5, V10, V15, V20) of whole bone or bone cavities of LSS were correlated most significantly with HT3+. An LSS mean dose of 23.5 Gy is associated with a 10 % risk of HT. Thus, recommended dose constraints to the LSS are V10 ≤ 80 % [7].

In our study we found LSS-V40 and gender were correlated with the risk of HT2+. Patients with LSS-V40 ≥ 60 % had higher rates of HT2+ than did patients with LSS-V40 < 60 % (38.3 %, 18/47 vs. 13 %, 6/46, p =0.005). The incidence of HT2+ was higher in female patients (46.9 %, 15/32) than in male patients (14.8 %, 9/61) (*p*-value =0.001). *Tait et al.* also found sex was the variable associated with any cardiac toxicity and pericardial effusion with multivariable logistic regression analysis in patients treated with chemoradiation therapy for esophageal carcinoma [23].

Given the evidence presented in this article for the existence of a sex-dependent hematologic toxicity difference, it is reasonable to seek explanations for potential mechanisms. Overall, it has been reported previously that women have a higher risk of myelotoxicity compared with men [2, 8, 12, 13]. This increased risk may likely be due to sex differences in pharmacokinetics and in pharmacodynamics [6, 24]. Makihara *et al.* found grade 4 neutropenia was frequently observed in females and gender could be considered as one of the important predictive factors associated with grade 4 neutropenia in patients receiving amrubicin monotherapy [15]. Milano *et al*. suggested that women were particularly prone to dihydropyrimidine dehydrogenase deficiency and there are data showing that the area under the curve, a measurement of plasma 5-FU level, is increased in women compared with men [14, 17]. Sloan *et al.* also reported women receiving 5-FU-based chemotherapy in a 5-day bolus schedule experience toxicity more frequently and with more severity than men [22].

In summary, there are no easy answers to the causes of increased hematologic toxicity of CRT in women and the reasons for this finding are likely multifactorial. Future studies should be directed to determine the mechanism behind the significant amount of women experiencing HT following CRT for rectal carcinoma. Based on our analysis, this specific finding was independent of the dose-volume relationship with toxicity and women did not demonstrate a significant difference in the dose of PBM compared with men. Potential analyses should investigate the rationale behind the lower tolerance of treatment in women versus men.

This study had some limitations because of the nature of retrospective studies, the small sample size and the small number of female patients. Our findings will need to be validated in a larger, prospectively collected group of data. In addition, all patients were treated with IMRT, and the results may not be applicable to patients being treated with conventional radiation therapy techniques. Finally, we contoured the entire bone as opposed to the actual bone marrow. However, there are currently no readily available imaging studies that would help delineate active bone marrow.

## Conclusion

In conclusion, this is the second study, to our knowledge, to identify dosimetric parameters associated with acute hematologic toxicity in rectal cancer patients undergoing chemoradiotherapy. Lumbosacral spine V40 was associated with clinically significant grade ≥ 2 hematologic toxicity. Keeping the lumbosacral spine V40 < 60 % was associated with a 13 % risk of grade ≥ 2 hematologic toxicity in rectal cancer patients undergoing concurrent chemoradiotherapy.

## Consent

Written informed consent was obtained from the patient for the publication of this report and any accompanying images.

## Abbreviations

LARC: Locally advanced rectal cancer
CRT: Chemoradiotherapy
HT: Hematologic toxicity
HT2+: Hematologic toxicity of grade ≥2
PBM: Pelvic bone marrow
LP: Lower pelvis
LSS: Lumbosacral spine
IMRT: Intensity-modulated radiation therapy
P-IMRT: Pelvic intensity modulated radiation therapy
